# Abbreviated HIV counselling and testing and enhanced referral to care in Uganda: a factorial randomised controlled trial

**DOI:** 10.1016/S2214-109X(13)70067-6

**Published:** 2013-08-23

**Authors:** Rhoda K Wanyenze, Moses R Kamya, Robin Fatch, Harriet Mayanja-Kizza, Steven Baveewo, Gregory Szekeres, David R Bangsberg, Thomas Coates, Judith A Hahn

**Affiliations:** Department of Disease Control and Environmental Health, Makerere University School of Public Health, Kampala, Uganda; Department of Medicine, Makerere University School of Medicine, Kampala, Uganda; Department of Medicine, San Francisco General Hospital, University of California San Francisco, San Francisco, CA, USA; Department of Medicine, Makerere University School of Medicine, Kampala, Uganda; Department of Medicine, Makerere University School of Medicine, Kampala, Uganda; Division of Infectious Diseases, University of California Los Angeles David Geff en School of Medicine, Los Angeles, CA, USA; Massachusetts General Hospital Center for Global Health and Harvard Medical School, Boston, MA, USA; Division of Infectious Diseases, University of California Los Angeles David Geff en School of Medicine, Los Angeles, CA, USA; Department of Medicine, San Francisco General Hospital, University of California San Francisco, San Francisco, CA, USA

## Abstract

**Background:**

HIV counselling and testing and linkage to care are crucial for successful HIV prevention and treatment. Abbreviated counselling could save time; however, its effect on HIV risk is uncertain and methods to improve linkage to care have not been studied.

**Methods:**

We did this factorial randomised controlled study at Mulago Hospital, Uganda. Participants were randomly assigned to abbreviated or traditional HIV counselling and testing; HIV-infected patients were randomly assigned to enhanced linkage to care or standard linkage to care. All study personnel except counsellors and the data officer were masked to study group assignment. Participants had structured interviews, given once every 3 months. We compared sexual risk behaviour by counselling strategy with a 6·5% non-inferiority margin. We used Cox proportional hazards analyses to compare HIV outcomes by linkage to care over 1 year and tested for interaction by sex. This trial is registered with ClinicalTrials.gov (NCT00648232).

**Findings:**

We enrolled 3415 participants; 1707 assigned to abbreviated counselling versus 1708 assigned to traditional. Unprotected sex with an HIV discordant or status unknown partner was similar in each group (232/823 [27·9%] *vs* 251/890 [28·2%], difference −0·3%, one-sided 95% CI 3·2). Loss to follow-up was lower for traditional counselling than for abbreviated counselling (adjusted hazard ratio [HR] 0·61, 95% CI 0·44–0·83). 1003 HIV-positive participants were assigned to enhanced linkage (n=504) or standard linkage to care (n=499). Linkage to care did not have a significant effect on mortality or receipt of co-trimoxazole. Time to treatment in men with CD4 cell counts of 250 cells per μL or fewer was lower for enhanced linkage versus standard linkage (adjusted HR 0·60, 95% CI 0·41–0·87) and time to HIV care was decreased among women (0·80, 0·66–0·96).

**Interpretation:**

Abbreviated HIV counselling and testing did not adversely affect risk behaviour. Linkage to care interventions might decrease time to enrolment in HIV care and antiretroviral treatment and thus might affect secondary HIV transmission and improve treatment outcomes.

**Funding:**

US National Institute of Mental Health.

## Introduction

High coverage of counselling and testing is crucial to the success of HIV prevention and treatment programmes.^1^ Uganda started the first voluntary HIV counselling and testing programme in Africa, expanding to all districts by 2005; however, access to testing could still be improved.^2^ Poor uptake of voluntary counselling and testing is a common problem worldwide^3,4^ and, in response, home-based and provider-initiated HIV testing and counselling have been adopted widely.^5–7^ Home-based testing refers to testing provided to entire communities in their homes by door-to-door visits or to household members of HIV-positive individuals. Provider-initiated testing refers to testing within a health-care setting that is initiated by a health provider to patients irrespective of their clinical diagnosis (as opposed to client-initiated or voluntary testing, which is instigated by the client). HIV testing in Uganda increased from 25% to 66% for women and from 21% to 45% for men between 2005 and 2011.^2,8^

A major challenge to the scale-up of provider-initiated HIV testing and counselling is the time needed to provide detailed counselling when faced with inadequate staffing and other competing health-care priorities. Therefore, the Ugandan Ministry of Health developed a brief HIV counselling protocol in 2006,^9^ almost entirely removing individualised risk assessment and pre-test counselling, while retaining general prevention messages such as risk reduction options, HIV serostatus disclosure, and partner testing.^9^ Abbreviated counselling might reduce the time burden on providers and increase the numbers of patients tested for HIV; however, whether it compromises the efficacy of sexual risk reduction afforded by traditional, individualised counselling is still unclear.^10–14^

After HIV diagnosis, rapid entry into care and initiation of antiretroviral therapy is needed to prevent secondary transmission and improve clinical outcomes; however, significant delays have been reported in prospective studies.^15,16^ Interventions to eliminate lags in entry into care could have an important effect on reducing secondary HIV transmission. The enhanced linkage to care intervention was an adaptation of the case-management linkage model, which resulted in a significantly higher rate of successful linkage to HIV care compared with passive referral.^17^

We investigated the effect of abbreviated versus traditional HIV counselling and testing on sexual risk behaviour and the effect of enhanced linkage to HIV care versus standard paper-based linkage on several HIV outcomes.

## Methods

### Study design and participants

We did this factorial, randomised controlled trial at Mulago Hospital, a large national referral and teaching hospital in Uganda. The first comparison assessed the effect of abbreviated HIV testing and counselling versus traditional testing and counselling on unprotected sex with an HIV discordant partner. The second comparison assessed the effect of enhanced linkage versus standard linkage on enrolment in HIV care, receipt of prophylaxis for opportunistic infections, receipt of antiretroviral therapy, and mortality.

Participants were patients in four medical inpatient wards and four medical outpatient clinics of Mulago Hospital.^15,16^ Eligibility criteria were: age 18 years or older, never been tested for HIV or tested negative more than 1 year before recruitment, lived within 25 km of Mulago Hospital, willing to have an HIV test, and sufficient cognitive ability. The institutional review boards of the University of California, Los Angeles (CA, USA); the University of California, San Francisco (CA, USA); and Makerere University School of Medicine (Kampala, Uganda) approved the study protocols and each participant provided written informed consent. An independent data and safety monitoring board met yearly to review study protocols, progress, and interim results. The full study protocol is available online.

### Randomisation and masking

We randomly allocated patients to abbreviated HIV testing and counselling or traditional HIV testing and counselling (1:1) in blocks of 100. Research counsellors were provided with the allocation through computer-generated randomisation after a baseline behavioural interview. To retain a roughly equal number of HIV-positive and HIV-negative participants for 1 year, HIV-negative participants were randomly assigned to follow-up or no follow-up (roughly 1:1). HIV-positive participants were randomly assigned to enhanced linkage to care or standard linkage to care (1:1); all were followed up for 1 year. These randomisations were computer-generated after HIV status was assessed. All study personnel except counsellors and the data officer were masked to study group assignment.

### Procedures

The abbreviated intervention was based on the Ugandan provider-initiated HIV testing and counselling protocols,^9^ and included a brief introduction to the benefits of HIV testing, the right to refuse testing, the importance of disclosure and partner testing, risk reduction options, and—in the event of a positive test result—available follow-up services. The participant was reassured that the test was being offered routinely and the session concluded with a question and answer period.

For the traditional intervention, we used the traditional HIV testing and counselling model that has been efficacious in east Africa and the Caribbean,^18^ endorsed by WHO, and widely implemented in voluntary counselling and testing centres in Uganda.^19^ The session included orientation, a personalised risk assessment, the development of a risk reduction plan appropriate for the patient's HIV status and social situation, and advice for status disclosure and partner referral. The session concluded with a question and answer period.

The enhanced linkage to care intervention was based on case-management referral and included: (1) counselling to reduce barriers to linkage to care; (2) assisted disclosure of HIV status to people who could provide social support; (3) introduction to the Mulago Immune Suppression Syndrome clinic and staff, and scheduling a follow-up appointment; and (4) a reminder via telephone or home visit (if no telephone) 1 week before the scheduled appointment. If the appointment was missed, up to four telephone call reminders or two home visits were attempted. The participants were allowed to opt out of HIV status disclosure. The standard linkage to care protocol involved an explanation of the available services, opening hours, and locations of the Immune Suppression Syndrome clinic and other nearby clinics. The participant was given information on paper about referral and the clinic.

Participants who were HIV-negative at baseline according to the Determine test (Abbott Laboratories; Abbott Park, IL, USA) were considered negative, and those who tested positive for both Determine and STAT-PAK (Chembio Diagnostics; Medford, NY, USA) tests were considered positive. Patients who tested positive by Determine but negative by STAT-PAK were tested with the Uni-Gold Recombigen test (Trinity Biotech, USA) and with the Amplicor DNA PCR qualitative test (Roche Diagnostics, Branchburg, NJ, USA).^20^ CD4 cell counts were taken at baseline, 6 months, and 12 months for HIV-positive participants. HIV testing was done at Mulago Hospital; CD4 testing was done at the Makerere University–Johns Hopkins University laboratory.

Participants completed a 30 min structured survey, given by an interviewer, once every 3 months for 1 year. Baseline surveys included participant demographics, HIV testing history, and concerns about HIV disclosure; follow-up surveys included assessment of HIV care and HIV status disclosure. Both baseline and follow-up interviews included detailed questions about up to five sexual partners in the past 3 months, including the perceived HIV status of each partner and frequency of condom use (always, sometimes, never) with each partner.

### Outcomes

The primary outcome variable for the comparison of HIV testing and counselling strategies was any unprotected sex with a potentially HIV-discordant partner (risky sexual behaviour) in the year after testing. A partner was defined as potentially HIV discordant if the participant had tested HIV-negative and reported that the partner was HIV-positive, if the participant had tested HIV-positive and reported that the partner was HIV-negative, or if the participant did not know the HIV status of the partner.

Outcome variables for the comparison of linkage to care were receipt of HIV care, receipt of co-trimoxazole for opportunistic infection prophylaxis, median time to start of antiretroviral therapy (in patients with baseline CD4 cell count ≤250 cells per mL), and 3-month and 1-year mortality. Receipt of HIV care was ascertained by asking if the participant had seen a medical provider in the past 3 months, and if so, which clinic they last visited. Clinics were classified post-hoc as HIV clinics or not (without knowledge of study group). Mortality was assessed by reports made by contacts encountered at home visits for tracking missing participants.

Covariates regarded as potential confounders included participant demographics, history of medical care, sexual behaviours, alcohol use, and site of recruitment. We created a household wealth index using principal components analysis—a method for reducing the number of correlated variables into a smaller number of dimensions—to group participants based on ownership of durable goods, housing quality, and available energy sources,^21^ and created three categories: low (0–40%), middle (41–80%), and high (81–100%).

### Statistical analysis

On the basis of our previous work, we estimated that recruiting 2000 participants would provide 1268–1384 people followed up for 1 year, assuming that 58% would test HIV-positive, mortality at 1-year would be 30–40% among those with HIV and 20% among those without HIV, and 5% would be lost to follow-up.^15^ Assuming that 40% would engage in risky sexual behaviour, we estimated that we would have 80% power to reject a null hypothesis of the inferiority of abbreviated HIV testing and counselling with a difference of 6·5% or less. During the study, HIV prevalence and mortality were lower than previously reported, consistent with trends of HIV prevalence in newly diagnosed patients admitted to hospital in Uganda. Therefore, to study the projected number of HIV-positive participants (638–754) for the comparison of access to care, we screened a larger number of people than originally planned, and stopped enrolment at the end of June, 2011, with 1003 HIV-positive people enrolled: 776 were in follow-up by July, 2012.

Planned analyses included non-inferiority tests of the proportion of participants reporting sexual risk behaviour in the year after HIV testing, in HIV-positive and HIV-negative participants. We would reject the null hypothesis of greater risky sexual behaviour in the abbreviated HIV testing and counselling group if the upper bound of the one-sided 95% CI for the difference in proportions was less than 6·5%. This analysis was done in both intention-to-treat and per-protocol populations using total duration of less than 30 min to define abbreviated HIV testing and counselling. We compared linkage to care approaches with Cox proportional hazards analyses, using a significance level of 0·048 to account for one interim analysis (done by JAH and RF in May, 2011, and shared with the data and safety monitoring board; all other study personnel remained masked to allocation). We did additional, post-hoc analyses to assess: (1) the effect of missing interviews on our analysis of HIV counselling and testing using multiple (five) imputations by chained equations, assuming data were missing at random; (2) the effect of adjusting for variables that were not equally distributed between randomised groups; (3) the effect of HIV counselling and testing strategy on sexual risk at each timepoint; (4) the effect of HIV counselling and testing strategy on study retention; (5) whether there was interaction between linkage to care group and sex, using the likelihood ratio test with a significance of p less than 0·10; and (6) the effect of HIV counselling and testing strategy on entry into HIV care, controlling for linkage to care group.

### Role of the funding source

The sponsor had no role in designing the study, analysing data, collecting data, interpreting the results, writing the report, or the decision to submit the paper for publication. The corresponding author had complete access to all the data.

## Results

**Figure 1:**
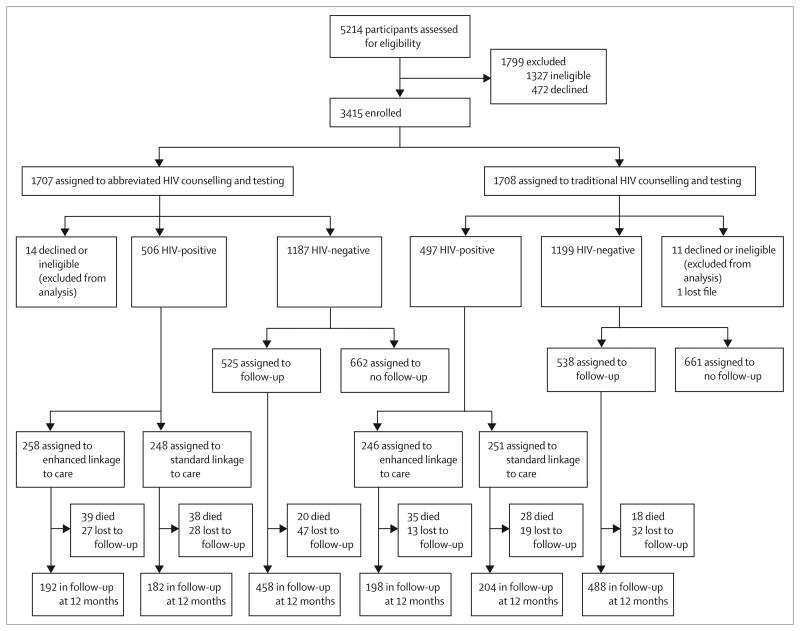
Trial profile Loss to follow-up includes participants who moved out of the area, who could not be located, and who did not complete their 12 month interview within the study follow-up window (within 1 month of due date).

From May, 2008, to June, 2011, we screened 5214 people. 1327 were ineligible and 472 declined to participate (figure 1); 3415 were enrolled. After assignment to a counselling strategy, 26 people were excluded from the study. Of the remaining 3389 participants, 1003 (30%) were HIV-positive and 2386 (70%) were HIV-negative. Of the HIV-negative participants, 1323 were randomly assigned to be excluded from follow-up, leaving 2066 HIV-negative and HIV-positive participants in follow-up. Of the 2066 people in follow-up, 178 (9%) died, 166 (8%) moved or were lost to follow-up, and 1722 (83%) were still in follow-up at 1 year.

Of the 2066 participants included in this analysis, 880 (43%) were men, and median age was 30 years (IQR 25–38). Table 1 shows baseline characteristics. Abbreviated HIV counselling and testing procedures took a median of 16 min (IQR 14–20), while the traditional sessions took a median of 47 min (IQR 45–50; p<0·0001).

Similar proportions of patients in the abbreviated versus traditional group (232/832 [27·9%] *vs* 251/890 [28·2%]), reported risky sexual behaviour at any follow-up interview; the upper bound of the one-sided 95% CI for the difference was 3·2%. Therefore, because the difference was less than 6·5%, we rejected the null hypothesis of inferiority of abbreviated HIV counselling and testing overall, as well as by month of follow-up and HIV status (table 2). These results did not differ qualitatively when we used multiple imputation to account for missed visits and loss to follow-up (other than death), when adjusted for household wealth, and when we did a per-protocol analysis (data not shown). We did post-hoc analyses of loss to follow-up by counselling strategy overall and stratified by HIV status. Loss to follow-up was significantly lower in the traditional group than in the abbreviated group overall (adjusted hazard ratio [HR] 0·61, 95% CI 0·44–0·83), as well as in patients with HIV (0·58, 0·38–0·90), while the groups did not differ significantly when considering only HIV-negative patients (0·66, 0·42–1·04).

1003 people were HIV-positive, of whom 378 (38%) were men, median age was 31 years (IQR 27–38), and median CD4 cell count was 283 cells per mL (IQR 130–463). Employment category and baseline CD4 cell count were significantly associated with linkage to care group in bivariate analysis; therefore, the HRs are adjusted for these variables, with CD4 cell count modelled as a continuous covariate (table 3).

**Figure 2:**
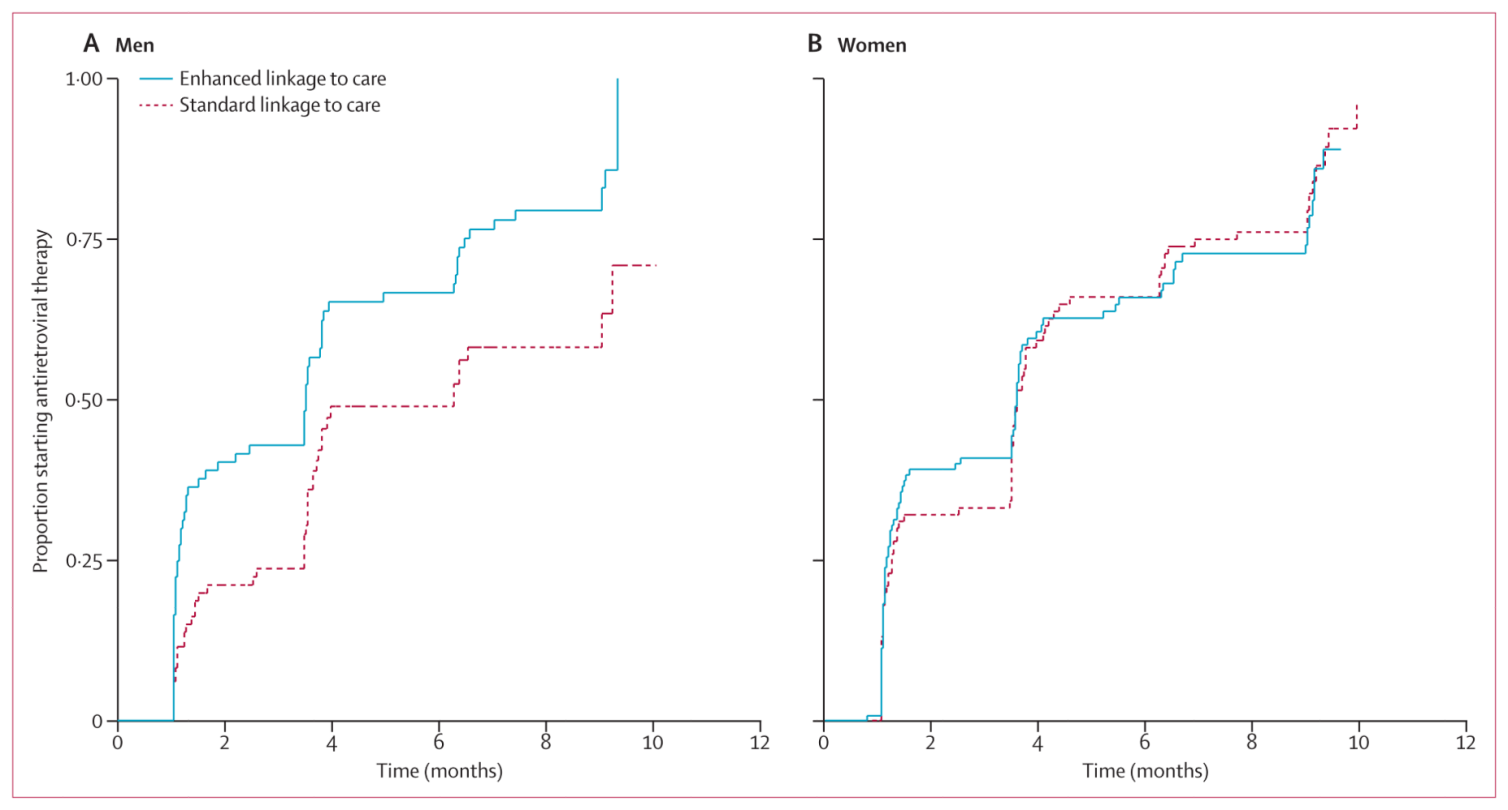
Kaplan-Meier curves of time to start of antiretroviral therapy in patients with CD4 cell count less than 250 cells per μL In men (A) and women (B). Adjusted for baseline CD4 cell count and employment.

We recorded no difference between linkage groups for mortality, receipt of prophylaxis for opportunistic infection, or receipt of HIV care (table 3). However, we report significant interactions (p<0·10) for two variables: time to HIV care was significantly different in women only (adjusted HR 0·80, 95% CI 0·66–0·96) and the effect of linkage to care on time to start of antiretroviral treatment was significantly different in men only (0·60, 0·41–0·87). In men with CD4 cell count of 250 cells per μL or less, median time to start of antiretroviral treatment was 107 days versus 192 days for enhanced linkage versus standard linkage (figure 2). In a post-hoc analysis, we recorded no difference between abbreviated and traditional HIV counselling and testing in time to HIV care, controlling for linkage to care group (data not shown).

## Discussion

HIV testing and linkage to care are essential for fighting the HIV epidemic, and evidence-based methods for increasing coverage of testing and accelerating linkage to care are needed. We report that abbreviated counselling was as effective as full length counselling in reducing sexual risk behaviour in both HIV-infected and uninfected individuals, and was roughly 30 min shorter in duration (panel). This finding has important implications for policy and practice in terms of scale-up of HIV counselling and testing services.^1^ Provider initiation increases uptake of HIV counselling and testing, and evidence suggests that it is no worse than other approaches to HIV testing in terms of ensuring informed consent, confidentiality, and counselling.^12,22,29^ Our findings extend these results by showing that an abbreviated counselling protocol can be used without fear of compromising the reductions in HIV risk behaviours afforded by detailed counselling. Adoption of abbreviated counselling could alleviate many of the burdens on medical professionals, while retaining value for patients. Time saved in counselling will probably reduce counsellor and provider effort and, eventually, the cost of provider-initiated HIV counselling and testing.^30^ Our study was not designed to assess the cost-effectiveness of provider-initiated HIV counselling and testing and additional assessment is needed. In addition, traditional counselling was associated with improved retention in the study among HIV-positive people, which may warrant further investigation.

Enhanced linkage significantly decreased time to start of antiretroviral treatment in men and entry into HIV care in women. Early entry into care and treatment can increase survival and decrease secondary HIV transmission.^1^ The difference by sex was surprising, but could be because, for women, enhanced linkage was helpful for initial clinic attendance, whereas for men, enhanced linkage did not increase the rate of getting to the clinic but instead served to retain them in clinic long enough to start treatment. Men access health care (for HIV or otherwise) less frequently and later than do women and have poorer retention in care than women.^31,32^ Thus, introducing men to the clinic could have an important effect on early treatment initiation.^1^ Enhanced linkage had no effect on receipt of prophylaxis for opportunistic infections; this finding is not surprising, since co-trimoxazole prophylaxis is cheap and nearly universal in HIV clinics in Uganda. The absence of an effect on survival might be because follow-up was only for 1 year; longer-term effects are plausible.

Our study had several limitations. The main outcomes—other than mortality—were self-assessed. Risky sexual behaviour at follow-up might have been under-reported, and those who received traditional, lengthier counselling might have been more likely to under-report risky behaviour because of social desirability. However, under-reporting in the traditional group would still support our conclusion that abbreviated counselling was no worse than traditional counselling in terms of sexual risk. To verify self-reported antiretroviral treatment, we reviewed patients' charts when treatment regimens were not specified in the study survey. For patients who received HIV care at Mulago Hospital, we verified that most (79%) agreed with the chart review to within 3 months. We might have underestimated the rate of receiving HIV care, because we asked about attending HIV care only at the participant's most recent medical visit. This misclassification could have resulted in a bias to the null.^33^ Another limitation was the different loss to follow-up in each counselling group. If greater loss to follow-up in the abbreviated counselling group was also associated with greater risky sexual behaviour, we could have incorrectly rejected inferiority. However, our results did not differ substantially when we used multiple imputation to account for missed visits. Another limitation is the use of a single site. However, many features of this setting—high HIV prevalence, widespread risky sexual behaviour, and persistently limited access to counselling, testing, care, and treatment services—are common to other health-care settings. The findings cannot be extended to home-based and community-based testing.

In conclusion, abbreviated HIV counselling and testing saves time and does not adversely affect risk behaviour; however, increased time spent with people who are HIV-positive might increase retention in care. Programmes that improve linkage to care can decrease the time to visiting an HIV clinic for women and time to start of HIV treatment for men. These findings suggest that shifting human resources previously devoted to traditional HIV risk reduction counselling to enhanced linkage to care could lead to more effective HIV prevention through improved treatment-mediated viral suppression without increasing HIV transmission risk behaviour.

## Figures and Tables

**Table 1:** Baseline characteristics

	Abbreviated HIV counselling and testing (n=1031)	Traditional HIV counselling and testing (n=1035)	Enhanced linkage to care (n=504)	Standard linkage to care (n=499)
HIV positive	506 (49%)	497 (48%)	504 (100%)	499 (100%)

Median CD4 cell count (IQR; cells per μL)	··	··	257 (126–427)	317 (140–503)

Median age (IQR; years)	30 (25–38)	30 (25–38)	31 (27–38)	32 (27–38)

Men	456 (44%)	424 (41%)	187 (37%)	191 (38%)

Marital status				
Married	442 (43%)	427 (41%)	218 (43%)	200 (40%)
Previously married	323 (31%)	337 (33%)	218 (43%)	217 (43%)
Never married	265 (26%)	271 (26%)	68 (13%)	82 (16%)
Missing data	1 (<1%)	0 (0%)	0 (0%)	0 (0%)

Education				
No formal education	55 (5%)	50 (5%)	31 (6%)	32 (6%)
Primary school	464 (45%)	477 (46%)	278 (55%)	253 (51%)
Secondary or tertiary school	510 (49%)	507 (49%)	194 (38%)	213 (43%)
Missing data	2 (<1%)	0 (0%)	0 (0%)	0 (0%)

Employment				
Labourer	344 (33%)	327 (32%)	159 (32%)	178 (36%)
Business, sales, or technical	438 (43%)	464 (45%)	204 (40%)	227 (45%)
Other	248 (24%)	244 (24%)	141 (28%)	94 (19%)

Household wealth				
High	142 (14%)	180 (17%)	59 (12%)	77 (15%)
Middle	390 (38%)	421 (41%)	197 (39%)	180 (36%)
Low	494 (48%)	432 (42%)	244 (48%)	241 (48%)
Missing data	5 (<1%)	2 (<1%)	4 (1%)	1 (<1%)

Frequency that household members go hungry				
Often	26 (3%)	27 (3%)	16 (3%)	14 (3%)
Sometimes	133 (13%)	111 (11%)	62 (12%)	72 (14%)
Seldom	103 (10%)	98 (9%)	62 (12%)	50 (10%)
Never	750 (73%)	778 (75%)	348 (69%)	352 (71%)
Missing data	19 (2%)	17 (2%)	16 (3%)	11 (2%)

Recruitment site				
Inpatient ward	196 (19%)	206 (20%)	90 (18%)	95 (19%)
Outpatient ward	695 (67%)	708 (68%)	325 (64%)	325 (65%)
Emergency or casualty department	140 (14%)	121 (12%)	89 (18%)	79 (16%)

Previous treatment in a clinic				
None	746 (72%)	748 (72%)	372 (74%)	364 (73%)
>12 months ago	17 (2%)	13 (1%)	8 (2%)	2 (<1%)
≤12 months ago	123 (12%)	135 (13%)	60 (12%)	65 (13%)
Don't know or declined	145 (14%)	139 (13%)	64 (13%)	68 (14%)

Number of sexual partners in the past 12 months				
0	256 (25%)	241 (23%)	139 (28%)	117 (23%)
1	552 (54%)	560 (54%)	268 (53%)	263 (53%)
2–5	201 (19%)	214 (21%)	86 (17%)	107 (21%)
>5	16 (2%)	10 (1%)	6 (1%)	6 (1%)
Missing data	6 (1%)	10 (1%)	5 (1%)	6 (1%

Any unprotected sex in the past 3 months	510 (49%)	545 (53%)	248 (49%)	246 (49%)

Last alcohol use				
Never	459 (45%)	443 (43%)	186 (37%)	187 (37%)
>3 months ago	269 (26%)	278 (27%)	156 (31%)	141 (28%)
≤3 months ago	299 (29%)	308 (30%)	160 (32%)	168 (34%)
Missing data	4 (<1%)	6 (1%)	2 (<1%)	3 (1%)

Data are n (%) or median (IQR).

**Table 2:** Sexual risk behaviours in participants still in follow-up at 12 months

	Abbreviated HIV counselling and testing	Traditional HIV counselling and testing	Difference (95% one-sided upper CI)
**All participants**			

At any follow-up visit	232/832 (27·9%)	251/890 (28·2%)	−0·3% (3·2%)
At 3 month visit	105/814 (12·9%)	111/861 (12·9%)	0·0% (2·7%)
At 6 month visit	99/808 (12·3%)	114/856 (13·3%)	−1·1% (1·6%)
At 9 month visit	96/802 (12·0%)	104/870 (12·0%)	0·0% (2·6%)
At 12 month visit	98/832 (11·8%)	101/890 (11·3%)	0·4% (3·0%)

**HIV-positive participants**			

At any follow-up visit	100/374 (26·7%)	114/402 (28·4%)	−1·6% (3·7%)
At 3 month visit	45/363 (12·4%)	50/386 (13·0%)	−0·6% (3·4%)
At 6 month visit	44/364 (12·1%)	50/382 (13·1%)	−1·0% (3·0%)
At 9 month visit	44/361 (12·2%)	50/393 (12·7%)	−0·5% (3·4%)
At 12 month visit	40/374 (10·7%)	49/402 (12·2%)	−1·5% (2·3%)

**HIV-negative participants**			

At any follow-up visit	132/458 (28·8%)	137/488 (28·1%)	0·7% (5·6%)
At 3 month visit	60/451 (13·3%)	61/475 (12·8%)	0·5% (4·1%)
At 6 month visit	55/444 (12·4%)	64/474 (13·5%)	−1·1% (2·5%)
At 9 month visit	52/441 (11·8%)	54/477 (11·3%)	0·5% (3·9%)
At 12 month visit	58/458 (12·7%)	52/488 (10·7%)	2·0% (5·4%)

Data are n/N (%) unless otherwise stated. Sexual risk behaviour is defined as any unprotected intercourse with a potentially HIV discordant partner in the 3 months before the interview.

**Table 3:** Cause-specific outcomes by linkage to care in HIV-positive participants

	Enhanced linkage to care group	Standard linkage to care group	Adjusted* hazard ratio (95% CI)	p value
Early death (≤90 days)	46/483 (10%)	36/482 (7%)	0·87 (0·56–1·35)	0·53
Men	22/176 (13%)	20/184 (11%)	1·04 (0·56–1·93)	0·90
Women	24/307 (8%)	16/298 (5%)	075 (0·39–1·42)	0·37

Death (1 year)	74/483 (15%)	66/482 (14%)	0·97 (0·70–1·36)	0·88
Men	34/176 (19%)	37/184 (20%)	1·23 (0·77–1·97)	0·39
Women	40/307 (13%)	29/298 (10%)	0·80 (0·49–1·29)	0·36

Attended an HIV clinic at most recent medical visit	355/439 (81%)	357/446 (80%)	0·90 (0·77–1·04)	0·17
Men	117/155 (75%)	126/163 (77%)	1·10 (0·85–1·42)	0·48
Women	238/284 (84%)	231/283 (82%)	0·80 (0·66–0·96)	0·02

Received co-trimoxazole	416/439 (95%)	416/446 (93%)	0·95 (0·83–1·09)	0·48
Men	142/155 (92%)	152/163 (93%)	1·07 (0·85–1·35)	0·58
Women	274/284 (96%)	264/283 (93%)	0·89 (0·75–1·06)	0·19

Received antiretroviral therapy (baseline CD4 ≤250 cells per μL)	157/202 (78%)	130/183 (71%)	0·77 (0·60–0·97)	0·03
Men	66/81 (81%)	47/76 (62%)	0·60 (0·41–0·87)	0·008
Women	91/121 (75%)	83/107 (76%)	0·95 (0·70–1·30)	0·76

Interaction between participant sex and linkage to care group, with adjusted models, p≥0·10 for all except: attended HIV clinic (p=0·08) and received antiretroviral therapy (p=0·04).

* Adjusted for continuous baseline CD4 cell count, and stratified by baseline employment category.

## Panel: Research in context

### Systematic review

Because no previous studies had compared traditional and abbreviated HIV counselling and testing head to head before this study began in 2008, a systematic review was not possible. At the time of final data analysis, we reviewed articles that assessed access to HIV care and risk reduction after provider-initiated HIV testing and counselling, and models of provider-initiated counselling. We searched PubMed for articles published in English up to July 20, 2013. The search terms included: “provider initiated HIV counseling and testing”, “provider initiated HIV testing and counseling” “routine HIV counseling and testing”, “linkage to care”, “access to care”, “risk reduction”, “HIV risk behavior”, “brief HIV counseling”, “abbreviated HIV counseling”, and “detailed HIV counseling”. Our search returned 61 articles; nine of which assessed linkage to care or risk reduction with provider-initiated HIV testing and counselling or compared provider-initiated HIV testing and counselling with other approaches. We excluded articles that described only uptake of testing or HIV testing and counselling processes. Three systematic reviews describe increases in uptake of testing after the introduction of provider-initiated schemes.^12,22,23^ Two articles^12,13^ reported increased condom use after provider-initiated HIV testing and counselling while access to care and other outcomes were mixed. Two studies^24,25^ compared access to care by testing mode (provider-initiated vs voluntary HIV testing and counselling) in people who tested HIV-positive; both reported lower access to care in those receiving provider-initiated HIV testing and counselling and one reported lower odds of starting treatment and lower odds of retention in care for provider-initiated approaches.^24^ However, other articles reported increased access to care and treatment after provider-initiated HIV testing and counselling.^26–28^ Challenges to implementation of provider-initiated HIV testing and counselling—eg, logistics, data systems, human resources, and management—were reported.^23^ We found no articles that examined models of provider-initiated counselling specifically comparing the effectiveness of brief versus detailed HIV counselling.

### Interpretation

Studies show some moderate risk reduction with voluntary HIV testing and counselling, raising concerns that brief counselling through provider-initiated HIV testing and counselling could compromise these outcomes. However, no study had evaluated these outcomes for brief counselling in comparison to detailed counselling. To our knowledge, our trial is the first to assess brief versus detailed provider-initiated counselling. The findings show that unprotected sex with an HIV discordant or status unknown partner was similar with both approaches and that enhancing linkage to care reduced the time to start of treatment for men who were eligible for treatment and time to HIV care for women. These findings indicate a need to focus more on the linkage to care and retention after testing as opposed to providing detailed pre-test and post-test counselling in provider-initiated HIV testing and counselling.

## References

[1] Granich RM, Gilks CF, Dye C, De Cock KM, Williams BG (2009). Universal voluntary HIV testing with immediate antiretroviral therapy as a strategy for elimination of HIV transmission: a mathematical model. Lancet.

[2] Uganda Ministry of Health (2005). Uganda National Policy on HIV Counselling and Testing.

[3] De Cock KM, Marum E, Mbori-Ngacha D (2003). A serostatus-based approach to HIV/AIDS prevention and care in Africa. Lancet.

[4] Baggaley R, Hensen B, Ajose O (2012). From caution to urgency: the evolution of HIV testing and counselling in Africa. Bull World Health Organ.

[5] WHO/UNAIDS (2007). Guidance on provider-initiated HIV testing and counselling in health facilities.

[6] WHO/UNAIDS/UNICEF (2011). Global HIV/AIDS response: epidemic update and health sector progress towards universal access: progress report, 2011.

[7] Creek TL, Ntumy R, Seipone K (2007). Successful introduction of routine opt-out HIV testing in antenatal care in Botswana. J Acquir Immune Defic Syndr.

[8] Uganda Ministry of Health (2012). Uganda AIDS indicator survey 2011.

[9] Uganda Ministry of Health (2006). Routine HIV testing and counselling participant's manual.

[10] Fonner VA, Denison J, Kennedy CE, O'Reilly K, Sweat M (2012). Voluntary counseling and testing (VCT) for changing HIV-related risk behavior in developing countries. Cochrane Database Syst Rev.

[11] Denison JA, O'Reilly KR, Schmid GP, Kennedy CE, Sweat MD (2008). HIV voluntary counseling and testing and behavioral risk reduction in developing countries: a meta-analysis, 1990–2005. AIDS Behav.

[12] Kennedy CE, Fonner VA, Sweat MD, Okero FA, Baggaley R, O'Reilly KR (2012). Provider-initiated HIV testing and counseling in low- and middle-income countries: a systematic review. AIDS Behav.

[13] Kiene SM, Bateganya M, Wanyenze R, Lule H, Mayer K, Stein M (2009). Provider-initiated HIV testing in health care settings: should it include client-centered counselling?. Sahara J.

[14] Rennie S, Behets F (2006). Desperately seeking targets: the ethics of routine HIV testing in low-income countries. Bull World Health Organ.

[15] Wanyenze RK, Hahn JA, Liechty CA (2011). Linkage to HIV care and survival following inpatient HIV counseling and testing. AIDS Behav.

[16] Rosen S, Fox MP (2011). Retention in HIV care between testing and treatment in sub-Saharan Africa: a systematic review. PLoS Med.

[17] Gardner LI, Metsch LR, Anderson-Mahoney P, Antiretroviral Treatment and Access Study Study Group (2005). Efficacy of a brief case management intervention to link recently diagnosed HIV-infected persons to care. AIDS.

[18] The Voluntary HIV-1 Counseling and Testing Efficacy Study Group (2000). Efficacy of voluntary HIV-1 counselling and testing in individuals and couples in Kenya, Tanzania, and Trinidad: a randomised trial. Lancet.

[19] WHO (2003). Increasing access to HIV testing and counselling: report of a WHO consultation, 19–21 November 2002.

[20] Baveewo S, Kamya MR, Mayanja-Kizza H (2012). Potential for false positive HIV test results with the serial rapid HIV testing algorithm. BMC Res Notes.

[21] Filmer D, Pritchett LH (2001). Estimating wealth effects without expenditure data—or tears: an application to educational enrollments in states of India. Demography.

[22] Hensen B, Baggaley R, Wong VJ (2012). Universal voluntary HIV testing in antenatal care settings: a review of the contribution of provider-initiated testing & counselling. Trop Med Int Health.

[23] Roura M, Watson-Jones D, Kahawita TM, Ferguson L, Ross DA (2013). Provider-initiated testing and counselling programmes in sub-Saharan Africa: a systematic review of their operational implementation. AIDS.

[24] Topp SM, Li MS, Chipukuma JM (2012). Does provider-initiated counselling and testing (PITC) strengthen early diagnosis and treatment initiation? Results from an analysis of an urban cohort of HIV-positive patients in Lusaka, Zambia. J Int AIDS Soc.

[25] Dalal S, Lee CW, Farirai T (2011). Provider-initiated HIV testing and counseling: increased uptake in two public community health centers in South Africa and implications for scale-up. PLoS One.

[26] Lawn SD, Fraenzel A, Kranzer K, Caldwell J, Bekker LG, Wood R (2011). Provider-initiated HIV testing increases access of patients with HIV-associated tuberculosis to antiretroviral treatment. S Afr Med J.

[27] Weigel R, Kamthunzi P, Mwansambo C, Phiri S, Kazembe PN (2009). Effect of provider-initiated testing and counselling and integration of ART services on access to HIV diagnosis and treatment for children in Lilongwe, Malawi: a pre-post comparison. BMC Pediatr.

[28] McCollum ED, Preidis GA, Golitko CL (2011). Routine inpatient human immunodeficiency virus testing system increases access to pediatric human immunodeficiency virus care in sub-Saharan Africa. Pediatr Infect Dis J.

[29] Obermeyer CM, Neuman M, Desclaux A (2012). Associations between mode of HIV testing and consent, confidentiality, and referral: a comparative analysis in four African countries. PLoS Med.

[30] Menzies N, Abang B, Wanyenze R (2009). The costs and effectiveness of four HIV counseling and testing strategies in Uganda. AIDS.

[31] Kigozi IM, Dobkin LM, Martin JN (2009). Late-disease stage at presentation to an HIV clinic in the era of free antiretroviral therapy in Sub-Saharan Africa. J Acquir Immune Defic Syndr.

[32] Mills EJ, Beyrer C, Birungi J, Dybul MR (2012). Engaging men in prevention and care for HIV/AIDS in Africa. PLoS Med.

[33] Copeland KT, Checkoway H, McMichael AJ, Holbrook RH (1977). Bias due to misclassification in the estimation of relative risk. Am J Epidemiol.

